# Supplementary material for: Are carbon emissions trading and green financial instruments synergistic? -Comprehensive quantitative research based on content analysis
Source: PLoS One. 2024 Mar 7;19(3):e0298601. doi: 10.1371/journal.pone.0298601 (PMC10919601; doi:10.1371/journal.pone.0298601)
Supplement: S1 Data — (ZIP) [file pone.0298601.s001.zip › Data/地级市Z_F工作报告.zip!/╣π╢1⁄2/├»├√╩╨/└┤╫╘╣1⁄2╓┌║┼í╛┬φ┐╦╩2╛▌═°í┐_2010.docx]

各位代表：

我代表市人民政府向大会作政府工作报告，请予审议，并请政协委员和其他列席人员提出意见。

2009年工作回顾

过去的一年，面对严峻的经济形势，在市委的正确领导下，在市人大及其常委会和市政协的监督支持下，市政府坚决贯彻落实中央和省关于保增长、保民生、保稳定的决策部署，全力推进“三促进一保持”，千方百计保增长，危中求机调结构，脚踏实地惠民生，全市经济社会实现平稳较快发展，顺利完成了市十届人大四次会议确定的目标任务。

——经济增长目标顺利完成。全市生产总值1239.8亿元，比上年增长10.3%，高于全省平均增幅0.8个百分点。人均生产总值20118元，增长8.9%，高于全省平均增幅0.5个百分点。财政一般预算收入41.7亿元，增长14.68%，高于全省平均增幅4.45个百分点。

——项目建设取得重大进展。13项重点项目竣工投产，茂名石化油品质量升级改扩建工程等76项重点项目已开工建设，纳入省的重点项目超额完成年度投资计划。项目争取工作成绩显著，争取到中央扩大内需项目资金2.85亿元，有13个项目列入省政府第二批扩大内需项目，总投资334亿元，17个项目纳入《关于促进粤西地区振兴发展的指导意见》，计划总投资429亿元。项目前期工作取得重大突破，水东港区3万吨级进港航道工程、茂名港总体规划获得批复，包茂高速公路茂名段、热电厂7号机组、粤电煤炭码头等项目，已获省发改委同意开展前期工作。我市发展后劲显著增强。

——产业结构调整明显加快。地方工业发展强劲，规模以上地方工业增加值增长13.2%。工业经济效益居全省前列。第三产业增长16.3%，占GDP比重比上年提高3.8个百分点，对全市经济增长贡献率达72.7%。高新技术产业快速发展，企业专利申请量增长46.2%，科技创新能力有新提升。

——融资引资和征地拆迁工作取得重大突破。全年金融机构为我市融资80多亿元，相当于过去8年的总和。成功举办广州、深圳专场投资推介会，签约项目投资总额575.7亿元，目前市直已实施项目16项。政府土地储备大幅增加，市级土地储备达2万多亩。长期困扰我们的融资难、征地难问题得到有效化解。

——“双转移”取得显著成效。全市三个省级产业转移工业园在建及建成项目97个，总投资33亿元。珠海（茂名）产业转移园成功获取5亿元省竞争性产业转移扶持资金。电白和信宜产业转移工业园，分别被评为全省“双转移”考核优秀和良好等次。与中山合作建立首家省级农村劳动力培训转移就业基地，全年免费培训农村劳动力6.1万人，新增转移就业12.5万人。

——城市建设迈出重大步伐。茂名大道、红旗路、站前路新建改建工程竣工通车，市民期盼多年的西粤路等“半截路”开工建设。环水东湾新城建设取得重大进展，市民大道开工建设，碧桂园房地产项目落户新城区。茂名城市发展呈现出前所未有的活力和吸引力。

——人民生活有效改善。城镇居民人均可支配收入13160元，农民人均纯收入5784元，分别增长9.6%和7%。农村合作医疗实现全覆盖，农村社会养老保险试点启动，低保标准和五保户供养水平进一步提高，困难群众的生活切实改善。

——新的目标定位深入人心。以粤西会议的召开为标志，我市确立了新目标新定位。打造世界级石化产业基地、全省重要能源物流基地和特色现代农业基地，努力实现“四个转变”，已经成为全市干部群众的共识。

一年来，我们主要抓了以下工作：

一、全力以赴保增长。面对危机，我们想方设法扩大投资，出台了《茂名市重点投资项目审批“绿色通道”实施办法》，完善项目工作机制，加快推进重点项目建设。全年完成固定资产投资180.01亿元，增长23.5%。落实鼓励消费的各项政策，千方百计扩大城乡消费，汽车摩托车下乡、家电下乡产品销售总量分别位居全省第一和第三。全年社会消费品零售总额增长20.3%。出台了《关于促进我市房地产市场健康发展的若干意见》，引进知名房地产企业，房地产开发投资增长31.8%。把公积金个人住房贷款最高额度提高到45万元，商品房销售总额增长33.6%。积极扶持中小企业发展，扶持资金达5200多万元。引导和帮助企业拓展市场，全年外贸出口增长7.4%，有效稳定了外部需求。

二、加快建设现代产业体系。以茂名石化公司为依托，以石化工业区为主要载体，大力发展壮大石化产业。茂名石化180万吨/年蜡油加氢等装置建成投产，乙烯年产量首超百万吨，石化工业区新投产项目5个。尤为重要的是，茂名石化油品质量升级改扩建项目进入实施阶段，为我市建设世界级石化基地奠定了坚实的基础。加快技术改造和技术创新，电白水产品加工产业集群被认定为省级产业集群升级示范区。稳步发展现代农业，农业增加值增长5.1%。新增省级农业创新中心2家、名牌农产品3个、农业龙头企业2家。成功举办广东（茂名）首届现代农业博览会，我市申报“中国罗非鱼之都”通过专家评审。成功举办第十一届“两广十市”区域旅游合作联席会议，旅游总收入增长10.3%，荣获“2009年度国际最佳休闲城市”称号。加快发展现代物流业，茂名港货物吞吐量突破2000万吨，增长16.5%。

三、全力破解发展难题。抓住我国实行适度宽松货币政策的机遇，夯实融资平台，创新融资方式，突破了融资瓶颈。成立征地拆迁工作指挥部，完善工作机制，上下联动，初步改变了征地难的局面。深化共识，狠抓落实，强化问责，办事难现象有了较大改观。严厉打击扰乱经济秩序和社会治安的违法犯罪活动，发展软环境有效改善。

四、切实加强城乡建设。完成交通基础设施建设投资14.2亿元。洛湛铁路茂名段建设进入扫尾阶段，茂湛铁路茂名段开工建设。新建、改建公路1133公里，提前完成镇通建制村公路硬底化建设任务。制定并推进《茂名市城市总体规划》等一系列规划，实施城市建设技术标准，规范建设行为。加强城市管理，强力清拆违章建筑，有效整治脏乱差现象，加大绿化投入，城市面貌焕然一新。环水东湾新城路网建设项目、高地片区教育建设项目进入实施阶段，大洲岛开发、海底隧道等多个大型项目正在洽谈，博贺新港区进港公路、通用码头、航道疏浚工程等已具备开工条件。县城、中心镇和新农村建设取得新成绩。

五、大力发展县域经济。茂南区城郊型现代农业和特色工业加快发展，服务业增加值占GDP的53.3%。茂港区现代物流业发展较快，征地拆迁工作取得重大突破，财政一般预算收入增长20.4%。信宜市外贸出口增长44.2%，成功引入华润、紫金矿业等知名企业。高州市新上工业项目165个，农业综合开发国家级示范区加快建设。化州市新引进项目134个，建成投产项目72个，荣获“中国化橘红之乡”的美誉。电白县注重引导产业集聚发展，初步形成了产业集群效应。

六、加强节能减排和环境保护工作。关闭小火电30万千瓦。开展市辖区环境专项整治，关闭和取缔小造纸厂、小砖窑、小油厂94家。高州水库生态保护工作初见成效。信宜、高州、化州污水处理厂建成试通水。环境质量保持稳定，完成年度主要污染物减排任务，根本改变了市区重酸雨污染的状况，饮用水源水质达标率保持100%。创建林业生态市工作稳步推进。

七、深化各项改革。稳妥推进政府机构改革，启动了第四轮行政审批制度改革。事业单位开始推行绩效工资制度，新进人员实行公开招聘。茂名碳素厂等国有企业资产重组加快推进。车辆通行费年票制正式实施。全面启动集体林权制度改革。规范清理行政事业收费，全年减少涉及企业收费1.2亿元。

八、努力保障和改善民生。全市财政民生支出50.1亿元，占一般预算支出的51%，增长13.2%，为近年来最大增幅。新增城镇就业岗位5.3万个，就业形势保持稳定。进一步扩大社会保险覆盖面。扎实推进“规划到户、责任到人”扶贫开发工作。积极扶持少数民族聚居村发展经济。全市共解决1388户廉租房保障对象的住房困难问题，交付使用经济适用房218套。总数达120套的市区第三期廉租房已全部交付使用，第四、五、六期廉租房加快建设。充分发挥住房公积金的保障作用，全市共提取和放贷12.63亿元。扎实推进援川工作，累计完成投资9000多万元，已完工30个项目。

九、大力发展社会事业。积极发展科技事业，广泛开展产学研合作。促进义务教育均衡发展，改善农村学校办学条件。加快解决代课教师问题，中小学教师工资福利待遇基本实现“两相当”，教育支出增长16.5%。高中阶段教育毛入学率提高到68.8%，高考上本科线以上人数连续6年居全省第二。中等职业教育跻身全省前列。高等教育加快发展。加强文化基础设施和文化产业建设，文化遗产保护、文艺创作、新闻出版取得新成效，成功举办庆祝新中国成立六十周年和建市五十周年系列活动。加快发展卫生事业，农村薄弱卫生院改造如期推进，甲型H1N1流感防控工作扎实有效。成功举行第十一届市运会，体育事业取得新发展。社区服务不断加强。国防动员、民兵预备役、拥军优抚安置、人口计生、外事侨务、民族宗教、对台、殡改、统计、打私、气象、知识产权、防震、经济研究、方志、档案、驻外联络、人防等工作取得新成绩。老龄、妇女、儿童、助残等事业持续发展。

十、加强民主法制和政府自身建设。深入开展科学发展观学习实践活动，形成推动发展的强大动力。全面推进依法行政。自觉接受市人大及其常委会的监督，加强与市政协的民主协商和沟通，办理人大代表建议139件、政协提案311件。加强应急管理工作，提升应对突发事件能力。扎实推进信访维稳工作。加强社会治安综合治理，积极构建打防管控体系，社会秩序保持稳定。深入开展食品药品安全和安全生产工作。切实做好源头治腐、纠风专项治理、行政审批电子监察、审计监督、民主评议行风等工作，廉政建设和反腐败斗争取得新成效。

各位代表！更加可喜的是，在过去的一年，全市干部的精神状态、工作作风有了很大的改变。抓落实的意识、责任显著增强，抓落实的措施、办法明显增多，抓落实的效率、效果更加凸显，展现出了团结奋进、干事创业的良好风貌。这既是我们顺利完成去年目标任务的内在动力，更是推动茂名加快发展、科学发展的关键所在！

各位代表！过去的一年，我们困难重重；过去的一年，我们危中求进；过去的一年，我们开拓创新。成绩来之不易，成绩鼓舞人心。这是上级党委、政府和市委正确领导的结果，是全市人民共克时艰、奋力拼搏的结果。在此，我代表市人民政府，向全市人民，向中央、省驻茂单位和驻茂解放军、武警部队官兵，向各民主党派、人民团体以及社会各界人士，表示诚挚的感谢！向所有关心和支持茂名发展的香港、澳门特别行政区同胞、台湾同胞、海外侨胞和国际友人，表示衷心的感谢！

回顾一年来的政府工作，我们深深地体会到：必须树立信心、勇于面对，在困难面前，创造性地落实好各项政策措施；必须立足当前、着眼未来，在保增长的同时谋划长远，为未来发展创造有利条件；必须团结共进、同舟共济，发挥各级各方的积极性，整合资源形成合力；必须以人为本、关注民生，促进基本公共服务均等化，努力让全市人民共享发展成果；必须转变作风、狠抓落实，切实提高执行力，把各项措施和部署落到实处。这些体会弥足珍贵，对我们做好下一步的工作具有重大意义。

2010年工作安排

2010年是实施“十一五”规划的最后一年，是实现“三年大变化，十年大跨越”的开局之年。总的来看，今年我市发展环境可能好于去年。全球经济逐步复苏回暖，有望恢复性增长。中央继续实施扩大内需和改善民生的政策。我省经济回升向好，省委、省政府“加快粤西地区振兴发展”的战略决策给我市注入了强劲的发展动力。我市石化产业步入新一轮大发展，重大项目建设加紧推进，城镇化步伐加快，发展环境有效改善，经济发展动力不断增强，正面临着加快发展的重大机遇！但我们也要清醒地看到，国内外经济形势仍然复杂多变,我市经济社会发展还面临着重重困难、严峻挑战和巨大压力。重重困难主要体现在：经济发展速度不快、质量不高，投资规模偏小，经济内生动力仍然不足；地方工业总量不大，自主创新能力不强，现代产业体系尚未形成；县域经济薄弱；新增税源不多，财政收支压力增大；城乡建设任重道远；保障民生任务艰巨；抓落实的执行力和创造力有待提高。严峻挑战主要体现在：日趋激烈的区域竞争对我市构成了较大冲击，在各地竞相争夺资金、项目等生产要素的竞争中，我们经受着严峻的考验；沿海石化产业布点趋向分散，我市的石化产业优势有可能被弱化。巨大压力主要体现在：省委、省政府对我市提出的目标任务定位很高；人民群众对加快发展的期望很高；我市产业结构单一，不确定因素较多，保增长的压力相当大。因此，我们既要看到积极变化和有利条件，坚定发展信心，又要充分认识面临的困难和挑战，增强忧患意识，按照省的要求和市委的部署，以更加积极的态度、更加强烈的责任感、更加有效的举措，积极谋划和推动茂名科学发展。

今年经济社会发展的主要预期目标是：生产总值增长11%，单位生产总值能耗下降3.2%，财政一般预算收入按可比口径增长11%，社会消费品零售总额增长15%，全社会固定资产投资增长30%，外贸进出口增长3%（其中出口持平），居民消费价格总水平涨幅控制在3%左右，城镇登记失业率控制在4%以内，城镇居民人均可支配收入增长9%，农民人均纯收入增长7%。

做好今年的工作，必须围绕“一条主线”，抓好“六个着力点”。“一条主线”就是加快转变经济发展方式。“六个着力点”就是着力壮大经济规模；着力调整优化经济结构；着力推动城市转型；着力推进重大项目建设；着力推动区域协调发展；着力保障民生。

今年要重点抓好八个方面的工作：

一、加快转变经济发展方式，调整优化经济结构

扩规模、调结构、重创新是我市转变经济发展方式的基本要求，我们要加快建设现代产业体系，提升发展质量和水平。

做大做强石化产业。发展壮大石化产业是我市建设现代产业体系的主攻方向。要进一步完善产业发展规划。做好茂名石化公司炼油、乙烯厂区卫生防护距离内村庄居民搬迁安置工作，确保茂名石化油品质量升级改扩建工程顺利建设。加快推进60万吨/年对二甲苯等重大项目，启动200万吨/年乙烯扩建前期工作。大力引进石化中下游项目，建设完整的石化产业链。

积极培育新兴产业。研究制定新兴产业发展规划，加大扶持力度，重点发展新能源、新材料、生物医药、海洋生物、海洋工程、节能环保等新兴产业。支持茂名重力石化装备制造基地和茂港电力核电热交换器国产化等项目建设，发展壮大装备制造业。

加快传统产业转型升级。改造提升劳保手套、医药保健、竹编等传统产业，重点培育50个优势传统产业项目。强力推进矿产资源整合，做到适度、有序开发，加快广东粤电油页岩资源综合利用项目建设。争取开工建设热电厂7号机组。

推进园区建设和产业转移。按照“一区多园”的模式和循环经济理念，高标准规划建设茂名石化工业区、河西工业城和食品加工园区。用好5亿元扶持资金，放大资金效用。扩大园区面积，完善基础设施，增强产业承载力。营造“政策洼地”和“服务高地”，千方百计引进新企业，加快推进在建项目，鼓励支持现有企业上马新项目。支持各县（市、区）加快工业园区建设，努力承接发达地区产业转移，加快形成园区主导产业。

扶持中小企业发展。加快实施“中小企业成长工程”，争取有一批企业入选广东现代产业500强。加大品牌培育、申报力度，力争再创一批国家级、省级名牌产品和著名商标。支持和奖励中小企业上市融资。放宽市场准入，鼓励民间资本进入基础设施建设、公用事业、社会事业等领域，激活民间资本，扩大民间投资。

积极发展现代服务业。大力发展现代物流业，建设一批能源、建材、小商品、农副产品等专业性物流园区和市场，开工建设博贺新港区物流基地和粤电煤炭码头。加快发展现代商贸服务业、现代金融业。编制旅游业发展总体规划，整合资源，做好推介，加快基础设施建设，启动浮山岭风景名胜区建设，创建滨海、生态和文化旅游目的地。加强对房地产业的调控和引导，增加普通商品住房供应，鼓励自住和改善型购房需求。

抓好节能减排。坚持把节能减排作为转变经济发展方式的重要抓手，支持茂名石化上马节能减排工程，继续加强对小油厂、小化工厂和砖厂的整治。规划建设河西、茂南区、茂港区等3个污水处理厂，加快垃圾无害化处理设施建设，做好城乡垃圾回收处理。加强高州水库水源和生态保护，做好库区内的垃圾收集处理和污水处理。深入开展小东江流域环境整治。倡导低碳生活，努力保护好天蓝水清、林茂山绿的生态环境。

二、大力发展现代农业，推动农村加快发展

坚持把“三农”工作作为全部工作的重中之重，加快从传统农业大市向现代农业强市转变。

加强农业基础设施建设。统筹使用涉农建设资金，抓好农业标准化示范区和现代标准农田建设，强化基本农田保护。加快推进水利防灾减灾、农村饮水安全等工程的建设，动工兴建总投资16亿元的高州水库灌区续建配套和节水改造工程、东南部沿海地区调水工程，解决生产生活用水，增强农业综合生产能力。

提高农业产业化水平。稳定粮食生产。着力优化种养业结构，加快发展特色优势农产品。培育壮大龙头企业，发展农民专业合作经济组织，推进产业化经营。发展农产品深加工，引导农产品、海产品加工企业向食品加工园区集聚。抓好罗非鱼养殖、加工、出口一体化建设。增加科研投入，健全技术推广体系，提高农业科技贡献率。

促进农民增收。认真落实各项农业补贴政策。完善农村金融服务，解决农民创业贷款难问题。加大扶持力度，改善革命老区和贫困地区的生产生活条件，突出解决农村“一保五难”问题，促进农民脱贫致富。推进“规划到户、责任到人”扶贫开发工作，确保如期完成任务。促进农民就业创业，多渠道增加农民收入，努力让全市农民过上富裕安康的幸福生活！

三、积极推进城镇化，建设宜居城乡

城镇化是增强发展动力的重要依托。要以打造区域中心城市为目标，加快建设宜居宜业的现代化海滨城市。

抓好城市规划、建设和管理工作。尽快修编完善中心城区和环水东湾新城的控制性详细规划。优化提升中心城区，抓好路网畅通、城市整洁和生活配套工程。强化城乡规划监察管理，坚决拆除违章建筑，从严治理乱摆乱卖现象，整治交通秩序。整合优化城市管理职能，提升城市管理水平。建设市级绿道网、小良热带植物园，推进城区绿化美化，优化人居环境。加快环水东湾新城建设，修建海洋大道，完善环水东湾新城路网，推动高地片区、旦场片区房地产以及歌美海湿地公园的建设，确保市民大道在7月底之前建成通车。规划建设体育、文化、会展中心以及教育、医疗、市场等配套设施，完善城市功能。

推动县城、中心镇和乡村协调发展。建立镇村规划建设管理的长效机制。开展宜居城乡创建活动。抓好建制镇总体规划和村庄规划的编制工作，支持县城、中心镇和建制镇加快发展。放宽城镇户籍限制，促进农业转移人口逐步在城镇就业和落户。抓好市级村庄整治试点，推进村庄整治和“万村绿”大行动，开展农业生态示范村建设。

全力以赴推进“三旧”改造。加大旧城镇、旧厂房、旧村庄改造力度。市区重点做好河西旧厂房、旧村庄的改造，争取今年初见成效，用3年时间建成省“三旧”改造的示范点。各地要因地制宜，充分利用政策，加快推动“三旧”改造。

四、有效扩大内需，增强经济发展后劲

要把扩大内需与转变经济发展方式结合起来，拉动经济平稳较快地增长。

全力推进项目建设。精心谋划一批关系长远发展的重大项目，重点储备石化产业、传统特色产业以及关系民生的发展项目。加快重点项目征地和审批步伐，全力推进重点项目开工建设。强化对项目建设进度的督查，建立起以目标倒逼进度的有效机制。特别是要加快环水东湾新城和铁路、公路、博贺新港区的建设。扎实推进湛江机场迁建、广东西部沿海铁路、包茂高速公路茂名段等重点项目的前期工作。今年计划安排重点建设项目90项，总投资517亿元，确保年内完成投资117亿元。

有效扩大居民消费。切实增加城乡居民收入，改善居民消费预期。发展和完善消费信贷，改善消费环境。落实好家电、汽车、摩托车、建材下乡以及节能产品惠民工程、增加农机购置补贴、家电汽车以旧换新等消费政策，培育消费热点，促进城乡居民扩大消费。

努力开拓国内市场。组织、支持企业参加省内外商品展销会，支持企业建立和拓宽销售网络。引导企业对外开展经济技术和贸易合作，提高合作水平。

五、深化改革开放，增强经济发展动力

改革开放是实现富民强市的必由之路。要用改革的办法、开放的思维，努力破除体制机制障碍，全面提升发展的内生动力。

不断深化各类改革。全面推进市、县政府机构改革，确保今年上半年全部完成。深化行政审批制度改革，精简行政审批事项。推进事业单位分类改革、人事制度改革和绩效工资制度改革。开展富县强镇事权改革，加大对基层的政策支持力度。推动农村改革创新，加强土地承包和土地流转管理工作。加快集体林权制度改革，推进现代林业建设和国有林场改革。全面清查国有资产，深化国有企业改革。稳步推进水资源、污水和垃圾处理、矿产资源有偿使用等方面的收费改革。深化财税和投融资体制改革。强化依法治税，加强税收征管和非税收入管理，提高财政收入质量。优化支出结构，确保重点项目和民生支出，从严控制一般性支出。完善融资平台，抓好项目融资，优化投资机制。加强社会信用体系建设，发展信用担保机构。深入推进农信社改革和农村金融创新。开展政策性农业保险试点等保险工作。今年争取全社会新增融资100亿元以上。

千方百计招商引资。以产业招商、资源招商为重点，引进国内外大型石化企业、食品加工和矿产资源加工龙头企业到我市投资兴业。加大基础设施建设项目招商力度。办好广州（茂名）石化产业专家研讨会。与珠海联手开展产业转移工业园专题招商。加强对各县（市、区）招商引资的考核奖励。

积极扩大对外贸易。转变外经贸发展方式，加快培育新兴出口产业集群，引导传统出口产业转型升级，扩大自主品牌和高附加值产品出口，减少进出口货物外流。深度开发美欧日等传统市场，大力拓展东盟、南美、非洲等新兴市场。

六、发展科技教育文化事业，夯实经济发展方式转变的基础

做好科技、教育、文化和人才工作，是调整经济结构、转变经济发展方式的重要支撑。

大力发展科技事业。切实增加科技经费投入。深入推进产学研合作和科技服务体系建设，抓好国家火炬计划茂名石化产业基地、广东石化产业知识产权公共信息平台的建设。抓紧申报省高新技术产业开发区。做好知识产权的申请、保护和开发利用工作。争取建设国家危险化学品质量监督检验中心。培育发展高新技术企业和民营科技企业，引导企业积极进行技术改造和技术创新，提高创新能力。

优先发展教育事业。加快义务教育规范化学校建设，推进义务教育均衡发展。推进普及高中阶段教育，大力发展职业技术教育，确保如期实现普及高中阶段教育的工作目标。加快发展学前教育和特殊教育，鼓励和规范民办教育。加强教师队伍建设，全面推进素质教育。落实中小学教师工资福利待遇“两相当”，切实解决代课教师问题。提升高等教育发展水平。加快市区中小学校建设步伐。抓好茂名学院、第二技工学校扩建以及茂名职业技术学院、第二职业技术学校、卫生学校迁建工作，抓好市一中新校区建设。今年市级财政拟安排的教育支出比去年预算增长13.9%。

努力推进文化建设。深化文化体制改革，扶持公益性文化事业，发展文化产业，培育骨干企业，鼓励文艺精品创作，繁荣社会科学和特色文化。完善文化服务体系和市场体系。加强网络文化建设和管理。

狠抓人才强市工作。人才是我市加快发展的战略资源。要建立完善人才培养、引进、使用和激励的政策与机制，加强人才工作组织载体建设。充分挖掘和整合现有人才资源，特别要培养好使用好石化专业人才、高层次专业人才和特聘人才，努力创造“留得住、能干事、干成事”的良好环境。加强招才引技、招人引智工作，鼓励、支持各类人才来我市就业创业。

七、着力保障和改善民生，推进和谐社会建设

落实《广东省基本公共服务均等化规划纲要》精神，促进基本公共服务均等化，全力保障和改善民生。

千方百计扩大就业。就业是民生之本。我们要实施积极的就业政策，培育新的就业增长点，今年计划新增城镇就业岗位5万个。支持劳动者自谋职业、自主创业。鼓励高校毕业生到基层、中小企业就业和自主创业。抓好创业示范基地建设。大力开展就业援助。做好劳动保障维稳维权工作。出台农村劳动力培训和转移就业工作考核办法，加大培训转移力度，提高培训质量，计划新增转移就业13万人。今年市级财政拟继续安排1000万元，用于农村劳动力培训和转移就业工作。

完善社会保障体系。完善最低生活保障制度，扩大低保覆盖面。做好五保供养和救灾救济工作，健全社会救助体系。推进社会保险扩面征缴，抓好新型农村社会养老保险试点，加快社会保险各险种实现市级统筹。扩大失业保险基金支出范围试点。完善城镇居民基本医疗保险制度，推进医疗保险普通门诊统筹和城乡统筹工作。加快推动国有停业企业退出市场，今年市级财政拟安排1000万元用于市直困难企业处置和职工安置。努力解决困难企业退休人员和职工医疗保障问题。加强社保基金监管和退休人员社会化管理服务。加大保障性住房建设力度，力争再用两年时间基本解决全市符合条件的城镇低收入家庭住房困难问题，市区争取在今年内解决。扩大住房公积金制度覆盖面。

做好人口计生工作。落实人口计生管理目标责任制，坚决执行计生责任追究制度，严厉打击“两假”、“两非”，严肃查处党员干部违法生育行为。加大宣传力度，加强基层基础工作。完善计生利益导向机制，深入开展“两无”活动，稳定低生育水平，务必扭转我市计生工作全省倒数第一的落后局面。

大力发展社会事业。深化医药卫生体制改革，完善公共医疗卫生服务体系。做好重大传染病的防控工作。加快推进农村卫生院“改薄”工程。抓好新型农村合作医疗管理工作。完善社区卫生服务，健全医疗救助制度，深入推广高州市人民医院的经验。今年市级财政拟安排医疗卫生支出比去年预算增长10.8%。大力开展全民健身运动，发展体育事业。推进平安和谐社区建设。深化殡葬改革，规范殡葬服务管理。大力发展社会慈善事业。重视发展妇女儿童、老龄和残疾人事业。加强国防建设，做好“双拥”和民兵预备役工作。统筹做好审计、统计、经济研究、外事侨务、民族宗教、对台、打私、地志、档案、气象、地震、人防、驻外联络和红十字会等工作，推动社会全面进步。

加强和改善社会管理。抓好社会治安综合治理，推进社会管理“六项创新”工程，完善社会治安防控体系。对治安重点地区和突出问题进行整治，坚决打击地方涉黑涉恶势力和各种违法犯罪活动。做好信访工作，积极预防和妥善处置群体性事件。加强安全生产，坚决遏制重特大事故。强化食品药品安全监管。完善应急管理工作机制，提高应对突发公共事件能力。继续做好对口支援汶川县银杏乡恢复重建工作。

今年市级财政拟安排民生支出10.4亿元，比去年预算增长11.1%。

八、加强民主法制和政府建设，切实提高执行力

按照法治政府、责任政府、廉洁政府和服务政府的要求，进一步转变作风，切实提高行政效能。

加强民主法制建设。认真落实市人大及其常委会各项决议决定，自觉接受人大和政协的监督，主动听取各民主党派、工商联、无党派人士和各人民团体的意见建议。广泛接受社会公众和新闻舆论监督。实行科学民主决策，提高政府工作透明度。坚持依法行政，规范行政执法行为，加强政府法制工作。加强审计、监察工作，深入推进反腐倡廉建设。抓好基层政权建设，今年市级财政在继续对每个镇补助10万元，对村委会干部每人每月补助100元的基础上，将安排全市每个行政村6000元办公经费。

加强行政效能建设。两年内全部取消或下放市级行政审批事项，规范行政审批流程。建立和完善以首问负责制、限时办结制、责任追究制为重点的行政效能制度体系。抓好投资服务中心建设，加快电子政务建设，加强行政效能监察，提高办事效率，改善政务环境。严格执行问责制度，对执行上级决策部署不力、违反规定进行决策、不作为、乱作为等问题，予以问责。抓好公务员队伍的教育管理，建设学习型、服务型、创新型机关。

转变作风抓好落实。完成今年的目标任务，关键在于抓好落实。我们要在去年有较大改观的基础上，进一步锤炼作风，真抓实干。建立“以目标倒逼进度，时间倒逼程序，社会倒逼部门，下级倒逼上级，督查倒逼落实”的抓落实机制。建立目标责任考核制和岗位责任制，加大工作督办力度，把各项工作落到实处。各地各部门要加强协调配合，形成抓落实的强大合力。

今年还要做好“十二五”规划的编制工作，科学谋划我市新一轮大发展。

各位代表！今年的工作任务繁重、使命光荣。目标催人奋进，实干创造未来。让我们团结一致、锐意进取、扎实工作，努力推动茂名振兴发展！
